# Prevalence and persistence of transmitted drug resistance mutations in the German HIV-1 Seroconverter Study Cohort

**DOI:** 10.1371/journal.pone.0209605

**Published:** 2019-01-16

**Authors:** Patrycja Machnowska, Karolin Meixenberger, Daniel Schmidt, Heiko Jessen, Heribert Hillenbrand, Barbara Gunsenheimer-Bartmeyer, Osamah Hamouda, Claudia Kücherer, Norbert Bannert, the German HIV-1 Seroconverter Study Group

**Affiliations:** 1 Division of HIV and Other Retroviruses, Robert Koch Institute, Berlin, Germany; 2 Division of HIV/AIDS, STI and Blood-borne Infections, Robert Koch Institute, Berlin, Germany; 3 Medical Care Centre Jessen, Berlin, Germany; 4 Medical Care Centre City-Ost, Berlin, Germany; 5 Institute of Virology, Charité-Universitätsmedizin Berlin, Berlin, Germany; State of Israel Ministry of Health, ISRAEL

## Abstract

The prevalence of transmitted drug resistance (TDR) in antiretroviral therapy (ART)-naïve individuals remains stable in most developed countries despite a decrease in the prevalence of acquired drug resistance. This suggests that persistence and further transmission of HIV-1 that encodes transmitted drug resistance mutations (TDRMs) is occurring in ART-naïve individuals. In this study, we analysed the prevalence and persistence of TDRMs in the protease and reverse transcriptase-sequences of ART-naïve patients within the German HIV-1 Seroconverter Study Cohort who were infected between 1996 and 2017. The prevalence of TDRMs and baseline susceptibility to antiretroviral drugs were assessed using the Stanford HIVdb list and algorithm. Mean survival times of TDRMs were calculated by Kaplan-Meier analysis. The overall prevalence of TDR was 17.2% (95% CI 15.7–18.6, N = 466/2715). Transmitted NNRTI resistance was observed most frequently with 7.8% (95% CI 6.8–8.8), followed by NRTI resistance (5.0%, 95% CI 4.2–5.9) and PI resistance (2.8%, 95% CI 2.2–3.4). Total TDR (OR = 0.89, p = 0.034) and transmitted NRTI resistance (OR = 0.65, p = 0.000) decreased between 1996 and 2017 but has remained stable during the last decade. Viral susceptibility to NNRTIs (6.5%-6.9% for individual drugs) was mainly reduced, while <3% of the recommended NRTIs and PIs were affected. The longest mean survival times were calculated for the NNRTI mutations K103N (5.3 years, 95% CI 4.2–5.6) and E138A/G/K (8.0 years, 95% CI 5.8–10.2 / 7.9 years, 95% CI 5.4–10.3 / 6.7 years, 95% CI 6.7–6.7) and for the NRTI mutation M41L (6.4 years, 95% CI 6.0–6.7).The long persistence of single TDRMs indicates that onward transmission from ART-naïve individuals is the main cause for TDR in Germany. Transmitted NNRTI resistance was the most frequent TDR, showing simultaneously the highest impact on baseline ART susceptibility and on TDRMs with prolonged persistence. These results give cause for concern regarding the use of NNRTI in first-line regimens.

## Introduction

Globally, 36.7 million people are currently living with HIV. During the last 20 years, antiretroviral therapy (ART) has been massively scaled up and, as a result, the number of new HIV-infections has fallen by 35% since 2000 [1]. However, selective pressure from antiretroviral drugs promotes the emergence of HIV drug resistance mutations (DRM), which can compromise successful treatment. HIV-1 resistance is classified as 'primary' when there is no history of ART or as 'secondary', when mutations emerge during ART. However, both forms of resistance are associated with a delay in virologic suppression and an increased risk of virologic failure and onward transmission [2, 3]. In individuals receiving ART, drug resistant viruses evolve from wild-type viruses and coexist in a mixture of genetic variants [4]. As most drug resistance mutations impair viral fitness, replacement of the drug resistant virus with a wild-type variant in the absence of drug pressure can be advantageous for the pathogen [5–7]. Interruption of ART therefore rapidly leads to reemergence of fitter wild-type variants [8]. However, transmitted drug resistance (TDR) differs fundamentally from acquired drug resistance (ADR) because the wild-type virus is not archived in these individuals. The emergence of a wild-type variant therefore depends on the number of back mutations required, the relative fitness of mutant and wild-type virus, the rate of viral turnover and the presence of compensatory mutations [9]. The surveillance of transmitted drug resistance mutations (TDRMs) is generally based on the surveillance drug resistance mutations (SDRMs) list published by Bennett et al. in 2009, as recommended by the World Health Organization (WHO) [10]. However, the landscape of antiretrovirals is changing continuously and new mutations such as E138A/G/K/Q/R (that confers resistance to the NNRTI rilpivirine (RPV), approved in 2011) are therefore not covered by this list. Furthermore, mutations at subtype-specific polymorphic positions are excluded from the WHO SDRM list, despite a number of such mutations contributing substantially to drug resistance. In our study, the analysis of TDRMs was therefore based on the Stanford HIVdb SDRM list, which is updated regularly and includes all mutations in the WHO SDRM list apart from F53Y and V85I in protease [11, 12].

One aspect of TDR that is rarely studied is the long-term persistence of drug-resistant HIV-1 in ART-naïve individuals. TDRMs can persist significantly longer than acquired DRMs in the infected person, even in the absence of selective drug pressure [13, 14]. Information concerning the long-term persistence of TDRMs in the absence of drug pressure is limited because only a relatively small number of individuals in study cohorts elect to refuse ART upon diagnosis. However, the few existing studies do show that the periods of individual TDRM persistence are highly variable and that TDRMs can persist for several years [15–17]. This further supports the hypothesis that TDR is driven mainly by onward transmission from ART-naïve individuals rather than from patients with a history of ART [18–23].

The aim of our study was to gain insight into the prevalence of TDRMs in light of the changing landscape of HIV-1 subtypes in the German HIV-1 Seroconverter Study Cohort between 1996 and 2017 and to assess the proportion of study patients potentially at risk of ART-failure due to these TDRMs. We also analysed the long-term persistence of individual TDRMs in ART-naïve study patients. Compared to previously published analyses, our study has one of the largest sample sizes, allowing a more detailed insight into the persistence of TDRMs in the absence of selective drug pressure to be formed.

## Materials and methods

### Study population

The German HIV-1 Seroconverter Study is a nationwide, multicenter, open, prospective, long-term observational cohort study in which the date of HIV-1 seroconversion is known or reliably estimated for each individual taking part. The study was approved by the ethical committee of the Charité University Medicine Berlin and all study patients enrolled gave written informed consent. Study patients are categorized as (i) acute seroconverters if the HIV-1 seroconversion is confirmed by laboratory diagnostics defined as reactive or undetermined ELISA followed by incomplete Immunoblot or detectable HIV viral load or (ii) as documented seroconverters if a maximum interval of three years is given between the last negative and the first confirmed positive HIV-1 antibody test. The study design and inclusion criteria have been described previously in detail [24, 25]. The analyses presented here included study patients enrolled between 30.06.1997 and 18.01.2018.

### HIV-1 genotyping

Viral RNA was isolated from plasma samples using the QIAamp Viral RNA Mini Kit (Qiagen, Hilden, Germany) or by automatic extraction with the NucliSENS easyMAG platform (Biomerieux, Nürtingen, Germany). HIV-1 genotyping was performed on extracted RNA using the ViroSeq HIV-1 Genotyping System (Abbott, Wiesbaden, Germany) with subsequent population-based Sanger sequencing using the BigDye Terminator v1.1 Cycle Sequencing Kit (Thermo Fisher Scientific, Dreieich, Germany) or an inhouse HIV-1 genotyping assay covering the protease (PR) and reverse transcriptase (RT) with subsequent population-based Sanger sequencing using the BigDye Terminator v3.1 Cycle Sequencing Kit (Thermo Fisher Scientific, Dreieich, Germany) [26]. Sequence analysis was performed on an ABI Prism 310 capillary sequencer (Thermo Fisher Scientific, Germany). SeqMan Pro (Lasergene v10.0.1, DNASTAR, USA) was used to generate the consensus sequence. In addition, some HIV-1 sequences were provided by participating study centers. HIV-1 subtypes were assessed with the REGA HIV-1 subtyping tool Version 3.0 [27].

Sequences are available at GenBank with accession numbers MH470511 to MH472562 and as published previously [18, 28].

### Data analysis

All baseline HIV-1 genotyping results from ART-naïve study patients were included for the analysis of the TDR prevalence. TDRMs were defined as mutations that reduce viral susceptibility to antiretroviral drugs (potential low-level resistance, low-level resistance, intermediate resistance, high-level resistance) according to the SDRM list of the Stanford HIVdb algorithm version 8.4 [11, 12]. TDRMs for the three major drug classes were studied: nucleoside and nucleotide reverse transcriptase inhibitors (NRTIs), nonnucleoside reverse transcriptase inhibitors (NNRTIs) and protease inhibitors (PIs). A detailed list of TDRMs evaluated in this study can be found in S1 Table. In addition, analysis of TDR prevalence was performed according to the WHO SDRM list to enable comparison with TDR prevalence from other countries [10]. Time-trend analyses were performed with STATA SE15 (StataCorp LLC, Texas, USA).

In study patients with TDR, baseline susceptibility to PIs, NRTIs and NNRTIs was analysed using the Stanford HIVdb algorithm version 8.4 based on all mutations observed at baseline HIV-1 genotyping, including polymorphic positions [11].

If a TDRM was detected at baseline HIV-1 genotyping, all available HIV-1 genotyping results during the ART-naïve course of infection of the study patient were screened for the persistence of the TDRM. If the TDRM was lost during follow-up, the time point of loss was defined as the blood sampling date of the follow-up sample in which the TDRM was no longer detectable by population-based Sanger sequencing. TDRM persistence was calculated using a non-parametric Kaplan-Meier approach including either the duration until the TDRM was lost or the maximal observation time. A Kaplan-Meier plot was generated using Stata SE15 (StataCorp LLC, Texas, USA) showing the cumulative probability of TDRM loss versus the ART-free time since the date of infection. The mean survival time of TDRMs was estimated as the area under the survival curve and expressed with a 95% confidence interval (CI). If no loss of TDRM was observed, the maximal duration of observation was used as an approximation of the period of persistence of single TDRMs. Follow-up HIV-1 sequences (PR and RT) of each study patient were confirmed by a cluster analysis using TransmicBS [18] with a bootstrap of 0.95 and a mean pairwise patristic distance of 0.035 on a maximum likelihood phylogenetic tree generated with IQtree [29].

## Results

### Sample and patient characteristics

In total, baseline samples of 2715 HIV-1 positive, ART-naïve study patients in the German HIV-1 Seroconverter Study with known or reliably estimated date of seroconversion between 1996 and 2017 were successfully HIV-1 genotyped (Table 1). The study patients had a calculated date of seroconversion between the 05 January 1996 and 27 December 2017.

**Table 1:** Sample and patient characteristics in the German HIV-1 Seroconverter Study Cohort (1996–2017).

	**N (%)**
**Genotyped samples**	2715 (100)
**Gender**	
Male	2577 (94.9)
Female	129 (4.8)
Unknown	9 (0.3)
**Median age at seroconversion**	33 years
**Transmission**	
MSM	2404 (88.6)
HET	210 (7.7)
IDU	31 (1.1)
High prevalence country	16 (0.6)
Exposure at work	7 (0.3)
Blood products	1 (0.04)
Unknown	46 (1.7)
**Seroconversion status**	
Acute	1095 (40.3)
Documented	1620 (59.7)
**Time between last negative and first positive test**	
<12M	859 (53.0)
12M-18M	322 (19.9)
18M-24M	212 (13.1)
24M-36M	227 (14.0)
**HIV-1 subtype**	
B	2392 (88.1)
Non-B	323 (11.9)
**TDR**	
Sensitive	2249 (82.8)
Resistant	466 (17.2)
**TDR against**	
One drug class	425 (91.2)
Two drug classes	35 (7.5)
Three drug classes	6 (1.3)

N: Number; M: Months; MSM: Men who have sex with men; HET: Heterosexual contact; IDU: Intravenous drug users

Study patients were mainly male (94.9%), had a median age of 33.0 (IQR 27–39) years at seroconversion and were infected with HIV-1 subtype B (88.1%), followed by unique recombinant forms (URF, 2.7%) and subtype A1 (2.4%). 40.3% of study patients were acute and 59.7% documented seroconverters. 53.0% of the documented seroconverters had an estimated date of seroconversion with less than 12 months between the last negative and first confirmed positive HIV-1 test.

### Prevalence of TDR in the HIV-1 Seroconverter Study Cohort

**Fig 1:**
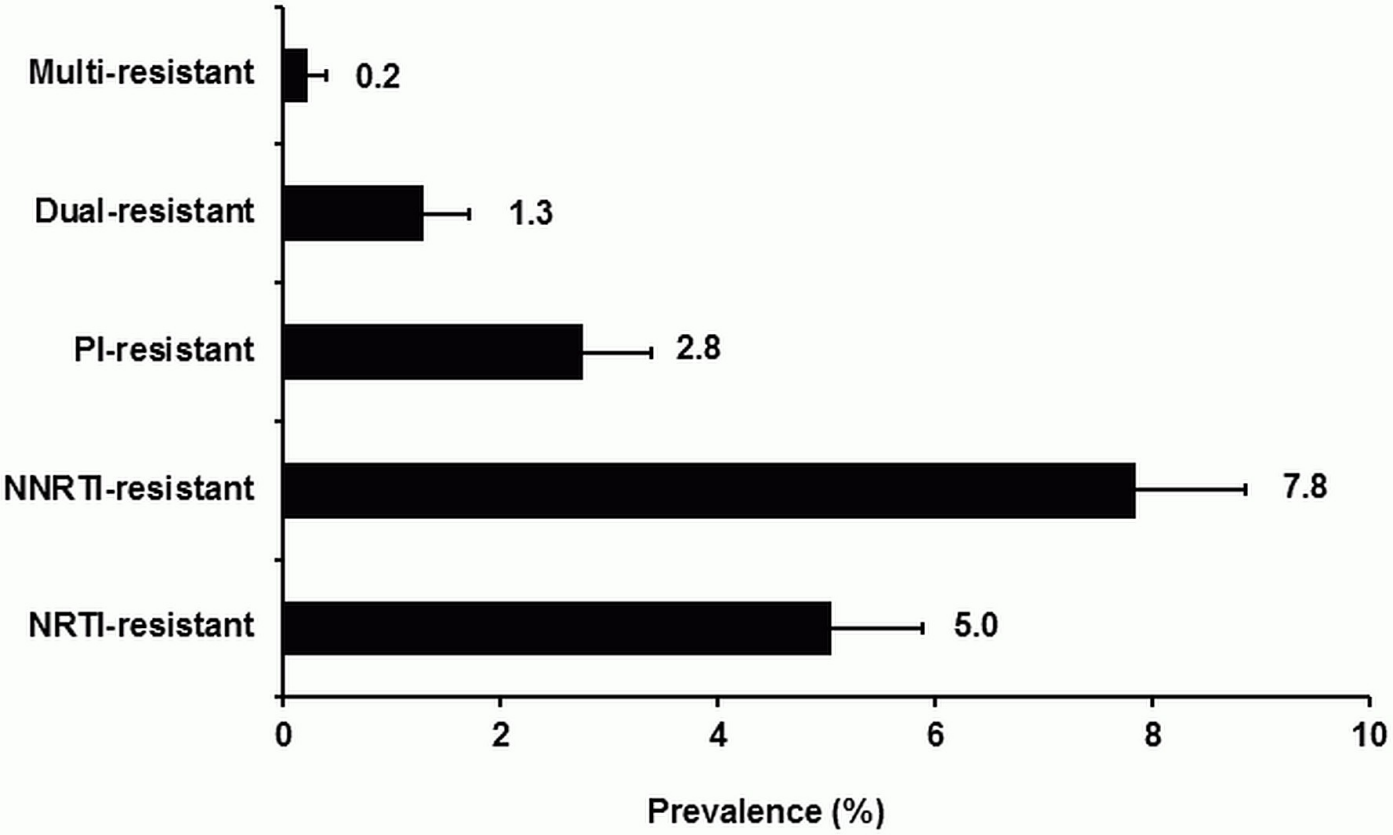
Overall prevalence of drug resistance classes in the German HIV-1 Seroconverter Study Cohort (1996–2017). Prevalence with 95% CI are given.

The overall prevalence of total TDR between 1996 and 2017 (year of seroconversion) was 17.2% (95% CI 15.7–18.6). Transmitted NNRTI resistance was most frequently observed in the study patients with 7.8% (95% CI 6.8–8.8), followed by 5.0% (95% CI 4.2–5.9) NRTI resistance and 2.8% (95% CI 2.2–3.4) PI resistance. Dual-class resistance was observed in 1.3% (95% CI 0.9–1.7) of the study patients, while only 0.2% (95% CI 0–0.4) of the study patients were infected with a triple-class resistant virus (Fig 1).

**Fig 2:**
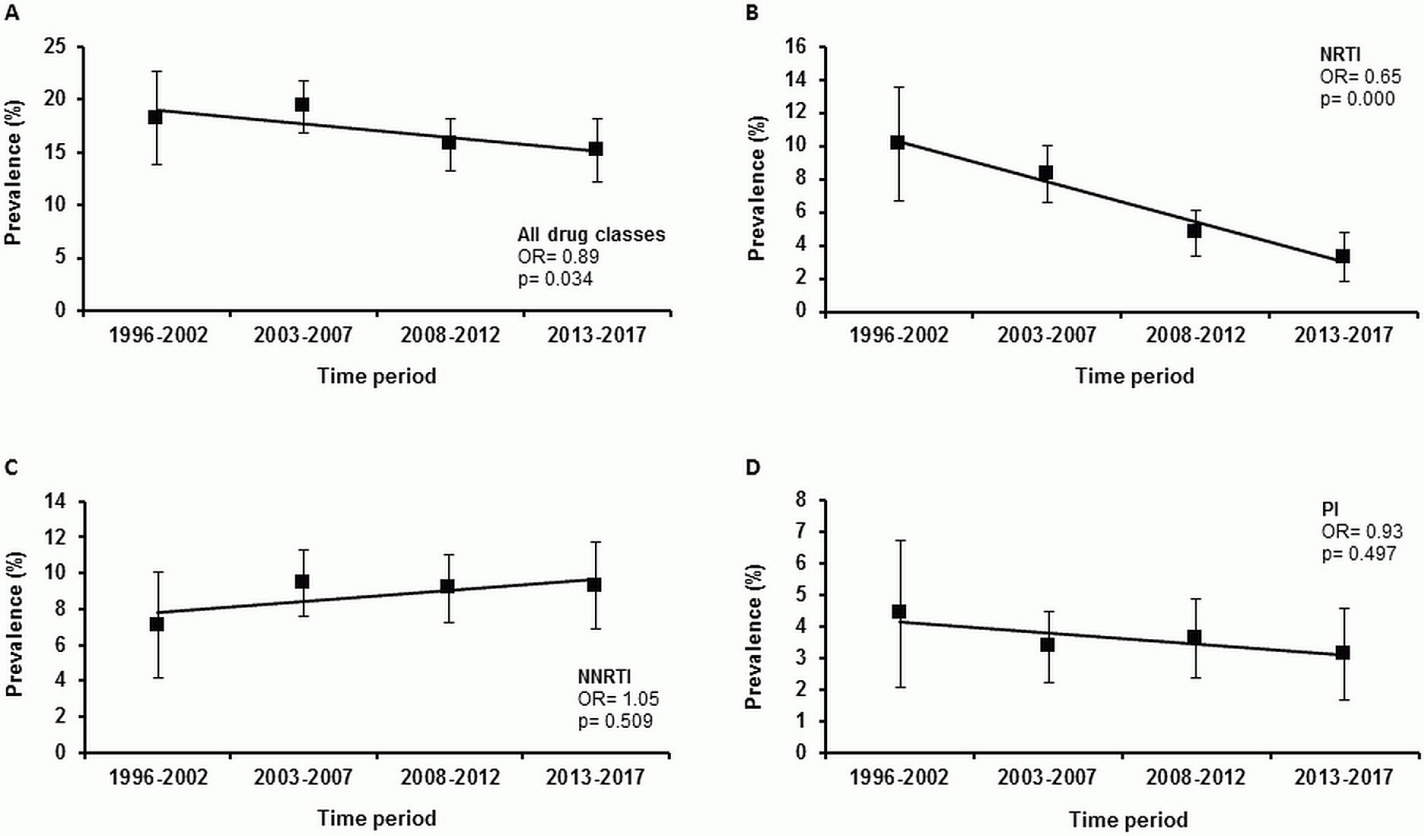
Prevalence trends of TDR in the German HIV-1 Seroconverter Study Cohort (1996–2017). N_(1996–2002)_ = 296, N_(2003–2007)_ = 985, N_(2008–2012)_ = 885, N_(2013–2017)_ = 549. A) Prevalence trend for total TDR. B) Prevalence trend for transmitted NRTI resistance. C) Prevalence trend for transmitted NNRTI resistance. D) Prevalence trend for transmitted PI resistance.

The prevalence of total TDR (OR = 0.89, p = 0.034, Fig 2A) and of transmitted NRTI resistance (OR = 0.65, p = 0.000, Fig 2B) decreased significantly in our study cohort between 1996 and 2017. In contrast, the prevalence of transmitted NNRTI (OR = 1.01, p = 0.509, Fig 2C) and PI (OR = 0.93, p = 0.497, Fig 2D) resistance remained stable between 1996 and 2017 without a significant trend.

Univariate logistic regression was performed for the following factors: country of origin, risk group, sex, age and HIV-1 subtype. Only the factors subtype B versus non-B subtype (OR = 2.90, p = 0.004) and German origin versus non-German origin (OR = 2.15, p = 0.005) were significantly associated with a higher risk of transmitted NRTI resistance.

If the analysis was restricted to the last ten year period between 2008 and 2017, no significant decline in the prevalence of total TDR (OR = 0.99, p = 0.714) or transmitted resistance to any drug class was observed (NRTI: OR = 0.95, p = 0.341; NNRTI: OR = 0.99, p = 0.848; PI: OR = 1.01, p = 0.863).

When applying the WHO SDRM list, the overall prevalence of total TDR was 10.6% (95% CI 9.5–11.8) in the entire study period (1996–2017) and 8.9% (95% CI 7.4–10.3) in more recent years (2008–2017). The prevalence for the different drug resistance classes were 5.0% (95% CI 4.2–5.8) NRTI resistance, 2.4% (95% CI 1.9–3.0) NNRTI resistance, 2.1% (95% CI 2.0–2.7) PI resistance, 0.8% (95% CI 0.5–1.1) dual-class resistance and 0.2% (95% CI 0.0–0.4) triple-class resistance.

### Susceptibility to antiretroviral drugs in the HIV-1 Seroconverter Study Cohort

**Fig 3:**
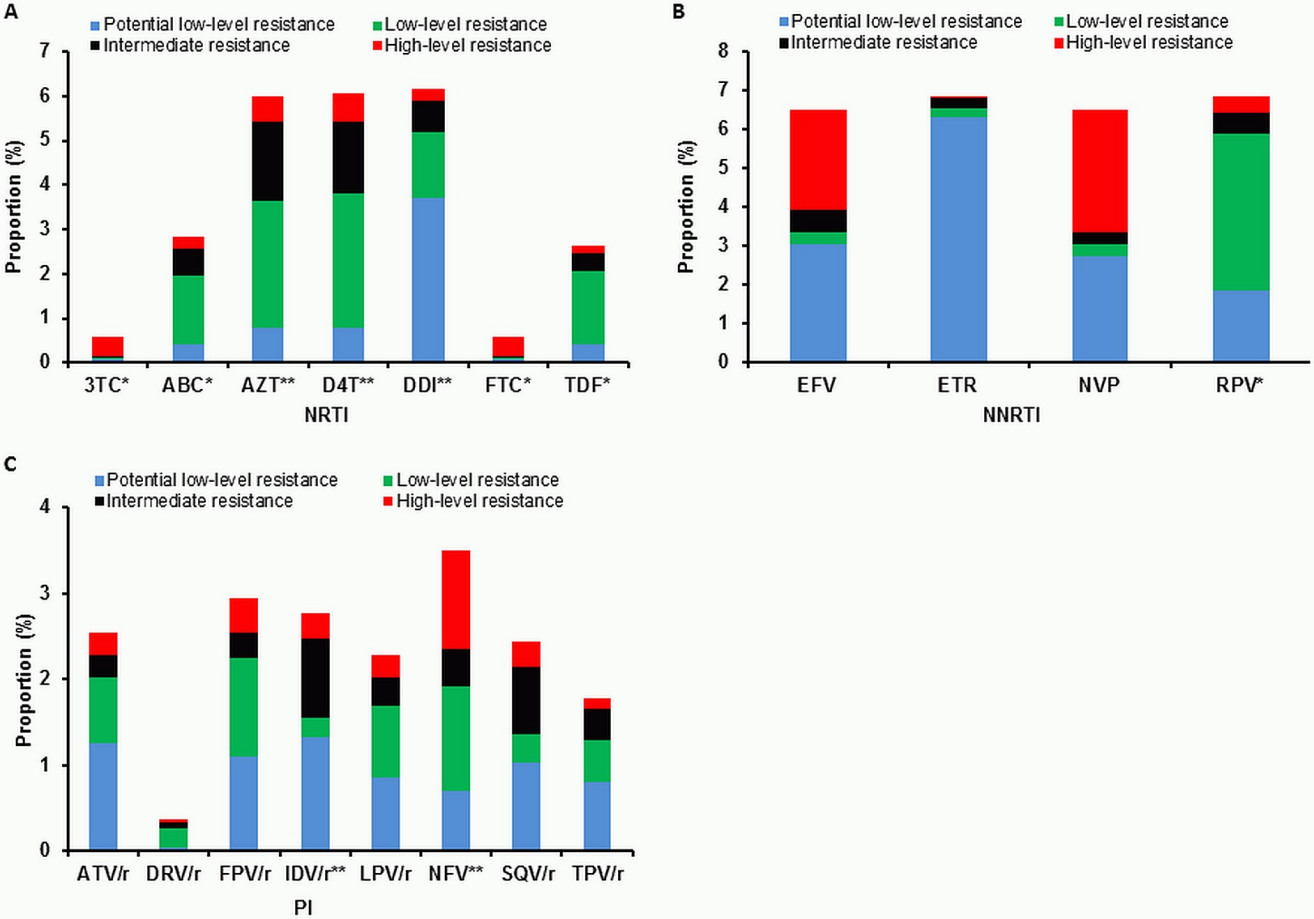
Predicted resistance to antiretroviral drugs in the German HIV-1 Seroconverter Study Cohort (1996–2017). *: first line-regimen; **: no longer recommended in European ART guidelines; /r: ritonavir-boosted; 3TC: lamivudine; ABC: abacavir; AZT: zidovudine; D4T: stavudine; DDI: didanosine; FTC: emtricitabine; TDF: tenofovir disoproxil fumarate; EFV: efavirenz; ETR: etravirine; NVP: nevirapine; RPV: rilpivirine; ATV: atazanavir; DRV: darunavir; FPV: fosamprenavir; IDV: indinavir; LPV: lopinavir; NFV: nelfinavir; SQV: saquinavir; TPV: tipranavir.

**Fig 4:**
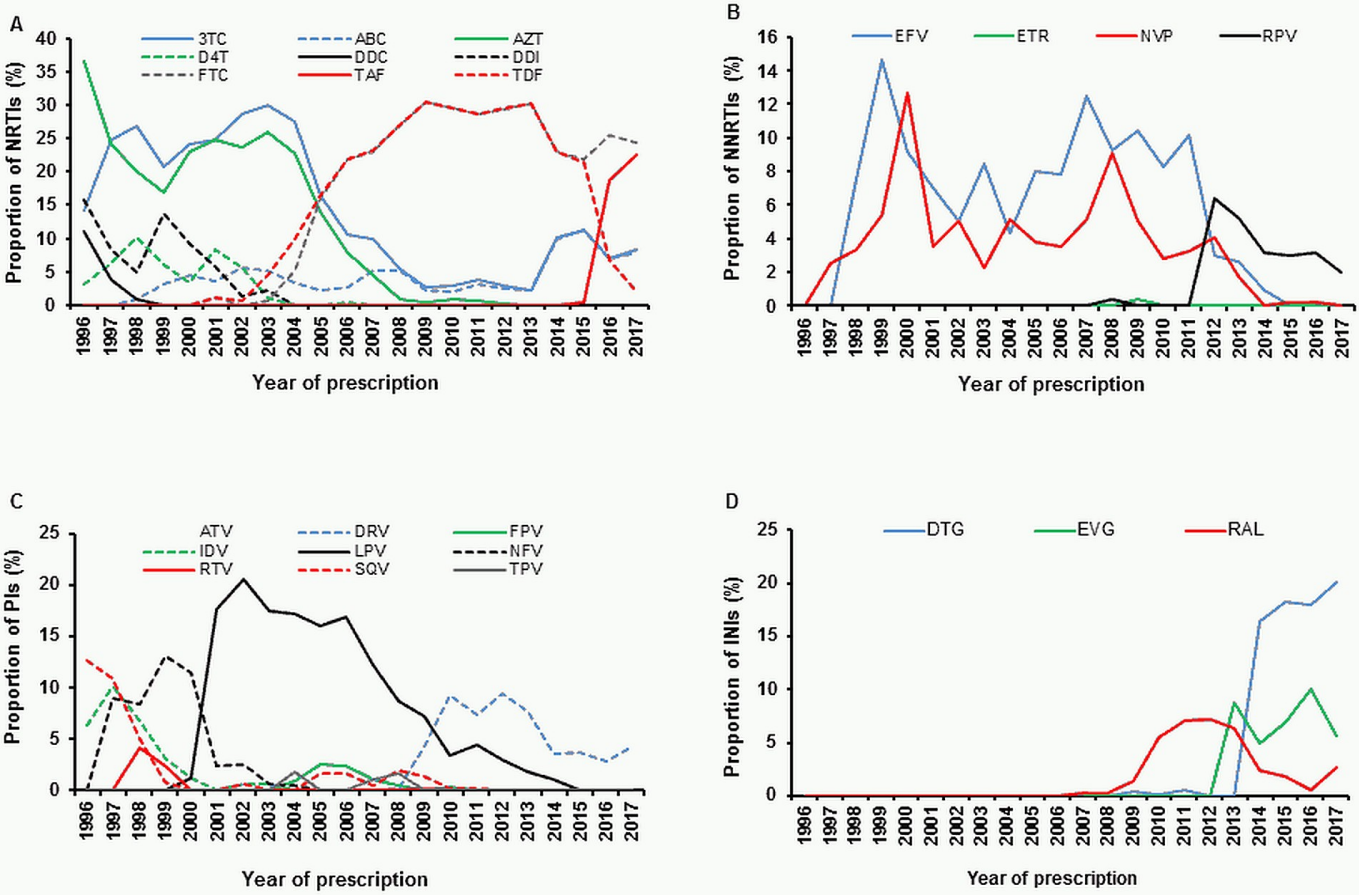
Drugs prescribed in first-line regimens in the German HIV-1 Seroconverter Study Cohort (1996–2017). A total of 2529 study patients started first-line ART between 1996 and 2017. The proportion of single drugs among all drugs prescribed in first line-regimens per year are given. N_(1996)_ = 63, N_(1997)_ = 157, N_(1998)_ = 119, N_(1999)_ = 130, N_(2000)_ = 87, N_(2001)_ = 85, N_(2002)_ = 160, N_(2003)_ = 177, N_(2004)_ = 233, N_(2005)_ = 236, N_(2006)_ = 255, N_(2007)_ = 392, N_(2008)_ = 475, N_(2009)_ = 497, N_(2010)_ = 596, N_(2011)_ = 592, N_(2012)_ = 639, N_(2013)_ = 684, N_(2014)_ = 543, N_(2015)_ = 565, N_(2016)_ = 505, N_(2017)_ = 303. A) NRTIs in first-line regimens. 3TC: lamivudine; ABC: abacavir; AZT: zidovudine; D4T: stavudine; DDC: zalcitabine; DDI: didanosine; FTC: emtricitabine; TAF: tenofovir alafenamide fumarate; TDF: tenofovir disoproxil fumarate. B) NNRTIs in first-line regimens. EFV: efavirenz; ETR: etravirine; NVP: nevirapine; RPV: rilpivirine. C) PIs in first-line regimens. ATV: atazanavir; DRV: darunavir; FPV: fosamprenavir; IDV: indinavir; LPV: lopinavir; NFV: nelfinavir; RTV: ritonavir; SQV: saquinavir. D) Integrase inhibitors (INIs) in first-line regimens. RAL: raltegravir; EVG: elvitegravir; DTG: dolutegravir.

The transmitted NRTI resistance mutations observed mostly affected the baseline susceptibility to didanosine (DDI), stavudine (d4T) and zidovudine (AZT), for which 6.2%, 6.1% and 6.0% of viruses, respectively, have been predicted to have at least a potential low-level resistance (Fig 3). However, due to toxicity, all three drugs are no longer recommended in the European ART guidelines [30] and have not been relevant for first-line regimens in our study cohort since 2003 (DDI, d4T) and 2008 (AZT) (Fig 4A). Predicted resistance to the NRTIs abacavir (ABC), emtricitabine (FTC), lamivudine (3TC) and tenofovir (TDF/TAF), which are still recommended, was limited to a maximum of 2.8% (Fig 3A). Since 2006, FTC and TDF/TAF have been the most commonly prescribed NRTIs in first-line regimens in our study cohort (Fig 4A).

The susceptibility to all currently available NNRTIs was highly affected by transmitted NNRTI resistance mutations, with a predicted resistance of 6.5% to 6.9%. For the second generation NNRTI etravirine (ETR), a high proportion of 6.3% was predicted to be potentially low-level resistant only (Fig 3B). However, ETR is not recommended for first-line regimens [30] and is therefore not frequently used in first-line regimens in our study cohort (Fig 4B). Resistance to the second generation NNRTI rilpivirine (RPV), which is the only NNRTI recommended for first-line regimens [30], was mainly caused by low-level resistant viruses carrying polymorphic mutations at position E138. Mutations E138A/G/K/R were found in 99 of 186 viruses (52.9%) showing RPV resistance, which account for 40% of all NNRTI-resistant viruses. Since its approval in 2011, RPV has become the most common NNRTI for first-line regimens prescribed in our study cohort (Fig 4B).

Transmitted PI resistance mutations had an overall low impact on the susceptibility to PIs. The highest resistance rate of 3.5% was observed to the first generation PI nelfinavir (NFV) (Fig 3C), which is no longer recommended in the European ART guidelines [30] and has not been relevant for first-line regimens in our study cohort since 2003 (Fig 4C). Among all PIs still in use [30], the susceptibility of fosamprenavir (FPV) was most affected with 2.9% predicted resistance. For darunavir (DRV), the only PI recommended for first-line regimens [30], the predicted resistance was only 0.4% (Fig 3C). Among PIs used in first-line regimens in our study cohort, DRV was also the most prescribed drug since 2010 (Fig 4C),

### Persistence of TDRMs in the study cohort

In total, 765 samples of 466 ART-naïve study patients were included in the analysis of persistence of TDRMs. The ART-naïve course of infection was followed in study patients in a range between 0 and 3730 days with a median of 258 days (95% CI 215–321). Follow-up samples were available for 146 of the 466 study patients (31.3%), whereas only the baseline sample was available for the remaining 320 study patients (68.7%). The median time between collections of follow-up samples was 1.1 years ranging between 10 and 1855 days. Detailed sample and patient characteristics can be found in Table 2. A total of 700 TDRMs were detected in the 466 baseline samples, of which only 37 TDRMs (5.3%) disappeared during follow-up sampling. 76.6% of viruses harbored one TDRM at baseline (singleton), 13.5% had two TDRMs and 9.7% had three or more TDRMs. The presence of three or more TDRMs was mainly due to the accumulation of Thymidine-Analogue-Mutations (TAMs: M41L, D67N, K70R, L210W, T215F/Y, T215 revertants and K219E/Q), which usually occur in mutation patterns [9]. Most viruses (91.2%) were resistant to one drug class, with only 7.5% and 1.3% showing resistance against two and three drug classes, respectively.

**Table 2:** Sample and patient characteristics in the analysis of persistence of TDRMs.

	N (%)
**Total**	466 (100)
**Gender**	
Male	442 (94.9)
Female	23 (4.9)
Unknown	1 (0.2)
**Transmission**	
MSM	416 (89.3)
HET	35 (7.5)
IDU	3 (0.6)
High prevalence country	3 (0.6)
Exposure at work	2 (0.4)
Unknown	7 (1.5)
**Seroconversion status**	
Acute	187 (40.1)
Documented	279 (56.9)
**Time between last negative and first positive test**	
<12M	153 (54.8)
12M-18M	51 (18.3)
18M-24M	36 (12.9)
24M-36M	39 (14.0)
**HIV-1 subtype**	
B	423 (90.8)
Non-B	34 (9.2)
**Number of mutations**	
One mutation	357 (76.6)
Two mutations	63 (13.5)
Three or more mutations	45 (9.7)

N: Number; M: Months; MSM: Men who have sex with men; HET: Heterosexual contact; IDU: intravenous drug users.

The most common TDRMs found in baseline samples were the T215 revertants, followed by E138A/G/K/Q/R, K103NS, V179DE and M41L (S1 Table). In total, TDRMs at 46 positions in PR and RT were analysed. For a number of TDRMs, no loss could be observed during the observation period (S1 Table) and, according to their maximal duration of observation, the following TDRMs seem to survive for lengthy periods in the absence of ART: the NRTI resistance mutations D67N (5.9 years), K70R (6.9 years), F77L (10.2 years), T215E (5.9 years) and K219Q (5.9 years), the NNRTI resistance mutations A98G (6.5 years) and Y188L (5.9 years) as well as the PI mutations I54L (5.9 years) and L90M (8.4 years) (S1 Table).

**Fig 5:**
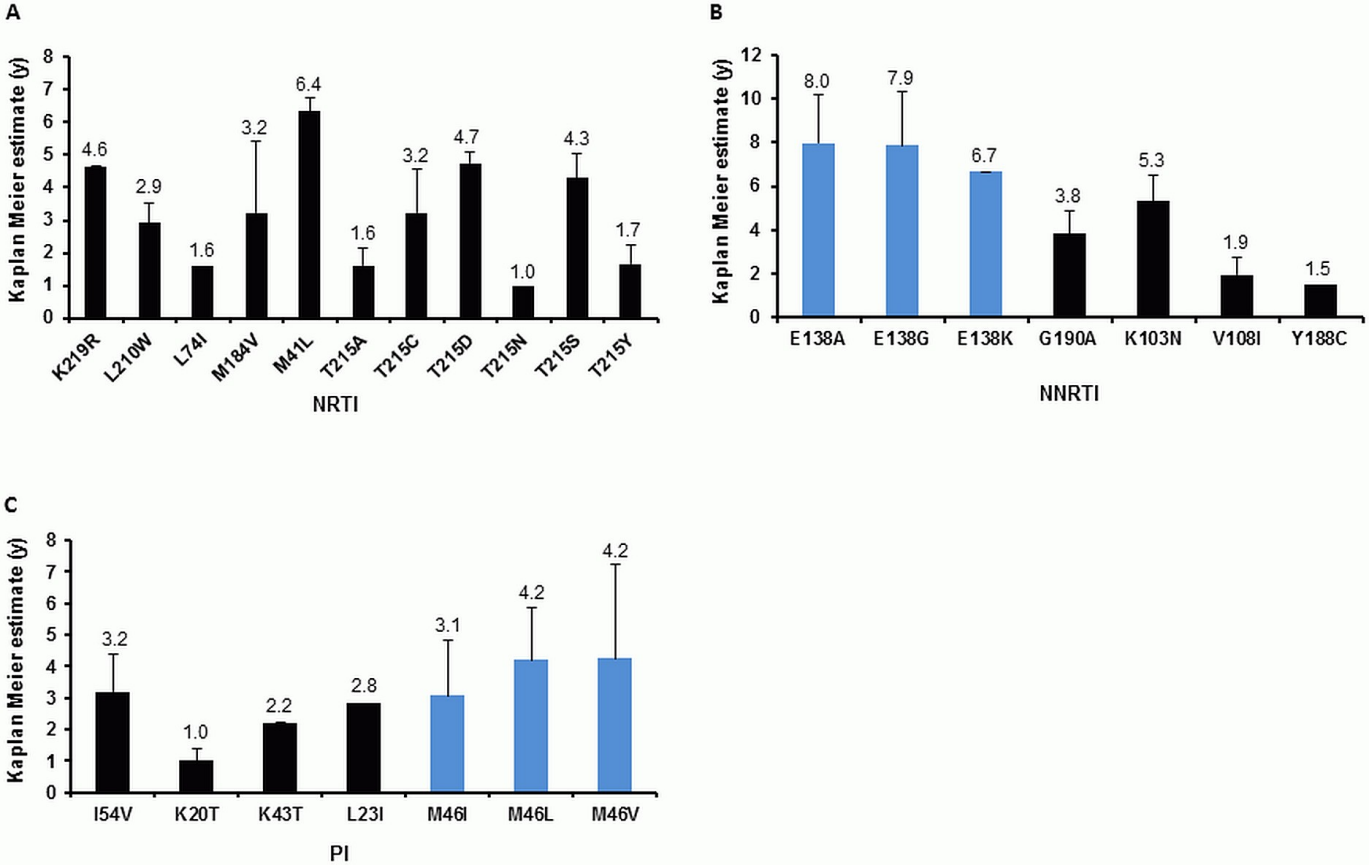
Mean survival times of TDRMs during ART-naïve course of infection. Mean Kaplan-Meier estimates with 95% CI are given. Different mutations occurring at the same position are shown in blue. A) TDRMs affecting NRTIs. B) TDRMs affecting NNRTIs. C) TDRMs affecting PIs.

A Kaplan-Meier analysis could be performed for 18 TDRMs (K20T, L23I, K43T, M46I/L/V, I54V, M41L, L74I, M184V, L210W, K219R, T215A/C/D/N/S and T215Y). Within the different drug classes, transmitted NRTI mutations persisted for a mean of 1.0 to 6.4 years, NNRTI mutations 1.5 to 8.0 years and PI mutations 1.0 to 5.1 years (Fig 5A–5C).

The NNRTI mutations at position E138 (E138A/G/K) had the longest mean Kaplan-Meier estimates (8.0 years, 95% CI 5.8–10.2 / 7.9 years, 95% CI 5.4–10.3 / 6.7 years, 95% CI 6.7–6.7) compared to all other TDRMs observed in this study cohort. Similarly, the NRTI resistance mutation M41L (6.4 years, 95% CI 6.0–6.7) and the NNRTI resistance mutation K103N (5.3 years, 95% CI 4.2–6.5) persisted for a long period. At NRTI position T215, the mean survival time varied with the specific substitution: T215D and T215S persisted for 4.7 (95% CI 4.4–5.1) and 4.2 (95% CI 3.5–5.1) years, respectively, while T215A and T215N persisted only for 1.6 (95% CI 1.1–2.2) and 1.0 (95% CI 1.0–1.0) years, respectively. Likewise, at PI position M46, the mean Kaplan-Meier estimate ranged from 3.1 to 4.2 years depending on the specific substitution. The shortest mean survival time of 1.0 year was determined for the PI resistance mutation K20T and the NRTI resistance mutation T215N (K20T 95% CI 0.6–1.4, T215N 95% CI 1.1–2.2).

## Discussion

In this study we present data on the prevalence of TDR between 1996 and 2017 and the persistence of single TDRMs in drug-naïve HIV-1 infected individuals enrolled in the German HIV-1 Seroconverter Study. With the large sample size of 2715 individuals included between 1996 and 2017, our data reliably display the long-term time trend of TDR in Germany. However, due to the high awareness of HIV-1 in MSM, which leads to frequent testing and therefore early diagnosis, this group is over-represented in our study cohort. At the same time, this also results in an under-representation of non-B subtypes, which are mainly detected in heterosexual individuals and individuals from countries of high HIV prevalence [31, 32] who are less likely to be eligible for our study due to their later diagnoses and missing information about the last negative test. Molecular surveillance data on HIV-1 in Germany with a higher percentage of non-MSM are available from recent years [19, 23]. However, it will become increasingly difficult in the future to acquire samples from patients with a known time point of infection and a long ART-naïve course of infection (as was possible with the German HIV-1 Seroconverter Study) because individuals often now commence virus-suppressing ART directly after diagnosis [30], making it difficult to address questions as the persistence of TDRMs.

Between 1996 and 2017, the overall prevalence of TDR in the German HIV-1 Seroconverter Study was 17.2% according to the Stanford HIVdb algorithm SDRM list [11, 12] and 10.6% according to the WHO SDRM list [10], which is in line with other European TDR studies covering comparable periods of time [33–37]. In particular, within the European surveillance program SPREAD, which includes newly infected individuals from 26 European countries, a TDR rate of around 9% has been observed since 2002 [38–41]. Likewise, an overall TDR prevalence of 11% was determined between 1996 and 2012 in the CASCADE collaboration in EuroCoord, which mostly includes HIV-1 seroconverters from Europe [42]. Different temporal trends for the prevalence of TDR and the single drug classes were observed in different study populations and over different time periods. In the present analysis we observed a decreasing trend in the overall prevalence of TDR between 1996 and 2017, which has not been apparent in the previous analysis covering the years between 1996 and 2010 [24]. The generally decreasing long-term but (in recent years) stable trend of TDR observed in our study cohort was also noticed in the large CASCADE analysis [42]. Genotypic resistance testing is therefore still important and should be continued (i) at the patient level before initiation of first-line ART and (ii) at the population level for molecular surveillance purposes. As the prevalence of ADR has been decreasing in developed countries for several years [34, 43], a main driver of the stable TDR rate appears to be onward transmission from ART-naïve individuals [35, 44–46]. We and others found that forward transmission occurs preferably early after infection when individuals are probably still unaware of their HIV-1 positivity and have high viral loads [18, 47–49]. Public health efforts should therefore be undertaken to facilitate earlier diagnosis and antiretroviral treatment to prevent onward transmission. Data from the German ClinSurv HIV Cohort showed that once initiated, ART is highly effective, with viral loads being successfully suppressed in 93% of study patients [50]. Therefore, the greatest potential for improvement indeed lies in the first two columns of the treatment cascade, namely early diagnosis and treatment, both of which currently have a coverage of 86% in Germany [51].

The proportion of transmitted NNRTI resistance in our study cohort was 7.8% according to the Stanford HIVdb algorithm SDRM list but only 2.4% according to the WHO SDRM list. The approximately 3-fold higher prevalence of NNRTI mutations based on the Stanford HIVdb algorithm SDRM list is mainly due to the polymorphic NNRTI mutations at position E138 that are not included in the WHO SDRM list. In our study cohort, NNRTI mutations at position E138 were found in 40% of all individuals with NNRTI resistance. Indeed, NNRTI resistance mutations affected the predicted baseline susceptibility to currently recommended first-line regimens the most. Current first-line regimens in Germany consist of a backbone of two NRTIs (TDF/TAF, FTC, ABC or 3TC) in combination with one NNRTI (RPV), one PI (DRV) or one INI (RAL, EVG, DTG) [30]. For the NNRTI RPV recommended for first-line regimens [30], the baseline susceptibility was predicted to be reduced in our study cohort, mainly due to the NNRTI-associated polymorphic resistance mutations E138A/G/K/R. These findings are in line with the results obtained by the large SPREAD study [41]. This underscores the need for HIV-1 genotypic resistance testing prior to the start of a first-line regimen. As the susceptibility of RPV is more often affected by baseline resistance than DRV or INIs [52], a combination of two NRTIs with DRV or RAL/EVG/DTG might be preferred. Indeed, our data concerning prescribed first-line regimens in the HIV-1 German Seroconverter Study Cohort showed that DTG has been most often used since 2014 (Fig 4D). It has recently been shown that the mutations E138K and M184I can mutually compensate for each other, resulting in restoration of viral replication capacity [53]. As the combination of RPV, FTC and TDF is approved as a single-tablet regimen (Eviplera), with RPV selecting for E138 mutations and FTC selecting for the M184I mutation, the emergence of E138 mutations, especially in combination with M184I, has to be monitored more closely in the future [54, 55].

Until now, studies into the persistence of TDRMs have been scarce. Nevertheless, three studies have performed survival analyses of single TDRMs or TDRM groups in patient cohorts of 75, 168 and 313 individuals [15–17]. In our study, 466 individuals with a known duration between infection and plasma sampling for HIV-1 genotyping could be included in the analysis of TDRM persistence in the absence of selective drug pressure. The longest mean survival times of approximately 8 years were observed for the NNRTI mutations E138A/G/K. Competition experiments have shown that E138A/G are the substitutions with the highest fitness, followed by E138R/K/Q, which is in line with the long mean persistence times found in our study [56]. Data on the *in vivo* persistence of E138 mutations have not so far been published, but NNRTI mutations generally have a low overall impact on viral fitness and are therefore expected to persist for a long time when transmitted [9, 16, 17]. Indeed, among the NNRTI mutations transmitted, the mutations K103N and G190A also showed long mean survival times of 5.3 (95% CI 4.2–6.5) and 3.8 years (95% CI 2.8–4.8), respectively, which agrees with the results from the three other cohort studies [15–17]. The K103N mutation, causing high resistance to the first generation NNRTI drugs NVP and EFV that are recommended for second-line regimens in Europe [30], was also frequently observed in our study cohort.

In general, transmitted PI resistance mutations tend to persist regardless of their reduced replication capacity due to fixation through fitness compensating polymorphisms [6, 9]. In concordance with the three other studies on the persistence of TDRMs [15–17], for most PI mutations (I54V, K43T, L23I, M46I/L/V) a long mean survival time between 2.2 and 4.2 years was found in our study cohort, with the exception of K20T that had a mean survival time of one (95% CI 0.4–1.2) year. We could not obtain a Kaplan-Meier estimate for L90M, but persistence of L90M was observed in one study patient followed for 8.4 years. This is in line with the data from Castro et al. and Yang et al. who observed persistence of L90M for up to 5.1 years [15, 17].

In our study cohort, the NRTI mutations M41L, L210W, T215C/D/S and K219R, which also occurred in high frequencies among the transmitted NRTI mutations, had calculated mean survival times of between 2.9 and 6.4 years. In contrast, the mean survival times determined for NRTI mutations T215Y/A/N and L74I, which were also found at a lower proportion among the transmitted NRTI mutations, ranged from 1.0 to 1.7 years. Although we could not obtain a Kaplan-Meier estimate for the mutation F77L, we did observe the mutation for 10.2 years in one study patient, the longest follow-up time for any TDRM in our cohort. Unfortunately, persistence data for this mutation are not available from other studies. Moreover, the mutations K70R and D67N were observed in one study patient each for 6.9 and 5.9 years, respectively. D67N has been shown in other studies to persist for a long time, while K70R has been shown to be lost rather quickly [15, 17]. However, the long persistence of K70R in our study was observed in a patient without additional TDRMs. It has been shown *in vitro* that the fitness cost of K70R is higher in combination with other mutations, possibly explaining its faster reversion in other *in vivo* cohort studies [5, 15, 17]. Overall, the persistence times of transmitted NRTI mutations also varied substantially but with a similar pattern in the three other studies addressing the persistence of TDRMs [15–17]. One exception was the long mean persistence time of 3.2 years (95% CI 1.9–5.4) of the M184V mutation in our study population, which is in contrast to the rapid loss observed in all other *in vivo* persistence studies [9, 15–17]. The most likely reason is that one study patient was included who lost the M184V mutation during an interval of 3.1 years between two plasma samplings. This leads to an overestimation of the mean survival time and is also reflected by the high 95% confidence interval of the mean Kaplan-Meier estimate. Despite the decreasing trend in transmitted NRTI resistance, which is probably due to decreasing acquired NRTI resistance, the overall prevalence of transmitted NRTIs in our study cohort was high and study patients of German origin infected with a subtype B virus had a higher risk of being infected with a NRTI-resistant strain. These findings are plausible given the potential for long persistence observed for several transmitted NRTI mutations and the further transmission soon after infection [18]. This is particularly true in light of the fact that subtype B is the most common HIV-1 subtype in Germany and that NRTIs were the first approved antiretroviral drugs in Germany, applied as monotherapy and therefore with a low genetic barrier to drug resistance, until the release of the first NNRTIs and PIs in 1996.

The results of our study and the studies of Castro et al. [15], Yang et al. [17] and Jain et al. [16] are not directly comparable because of differences in (i) the methodological approaches used to calculate survival times, (ii) the characteristics of the study populations and (iii) the sampling schemes and durations of observation. Nevertheless, a similar trend towards the survival times of single TDRMs is obvious in all studies. The fact that identical TDRMs with remarkably long persistence times have been observed in all four studies further supports the need of baseline genotypic resistance testing. Fortunately, according to the current treatment guidelines [30], only one drug—the NNRTI RPV—was significantly affected by long-term persisting TDRMs. Persisting transmitted NRTI mutations causing high levels of resistance affected NRTIs (DDI, d4T, AZT) that are no longer in use. Continued onward transmission of drug-resistant HIV-1 harboring persisting TDRMs might lead to the appearance of a new “wildtype” that is resistant to previously but not currently used drugs.

One major limitation of our study remains the irregular collection of plasma samples from study patients despite samples being generally requested on an annual basis and the median time between follow-up samples being only 0.7 years in our study cohort. An extended time between sampling can lead to an overestimation of the mean survival time, especially for mutations with short persistence as we have proposed for M184V. This might also be the case for mutations E138A, M46I, M46V and T215S, because for these TDRMs there was a period of more than 2.5 years between samples collected with and without the corresponding mutation.

A further limitation of our study is the fact that only the baseline sample was available for a large number of individuals (68.7%), lowering the calculated median observation time in person years compared to the longest observation time in years per TDRM. Small median observation times therefore do not predict short persistence times but rather reflect the high number of individuals without a follow-up sample. Short observation periods together with a presumed low prevalence [52] were also the reason for not analysing TDRMs affecting integrase inhibitors.

A final limitation of our study was the use of population-based Sanger sequencing for genotypic resistance testing, an approach unable to detect HIV-1 drug resistant variants at levels below approximately 20% [57]. It has previously been shown that using ultrasensitive assays reveals a much higher prevalence of TDRMs [58], as mutations with high fitness costs might in particular decay to low-levels by the time of HIV-1 diagnosis [59]. In our study, viruses with a single drug resistance mutation (singletons) were highly prevalent, and it is therefore conceivable that ultradeep sequencing might reveal the presence of further TDRMs, leading to an underestimation of the calculated survival rates as well as the TDR prevalence as other studies suggest [58, 60]. Nevertheless, despite using ultradeep sequencing other studies failed to find additional TDRMs [61, 62]. The impact of minority drug resistant variants on treatment success is therefore still a matter of debate as conflicting results have been observed in different studies [63].

Our data on TDR prevalence in Germany are in concordance with other studies from Europe. The stable prevalence of TDR in the last ten years in combination with the long persistence of single TDRMs found in this study indicates the onward transmission of TDR from ART-naïve individuals rather than from individuals with a history of ART as the main driver of TDR. Early diagnosis and antiretroviral treatment of HIV-1 infections are therefore required to prevent the spread of drug-resistant HIV-1. The high prevalence of NNRTI mutations at polymorphic position E138, which affect susceptibility to RPV and which had mean survival times of up to 8 years, already suggests that first-line regimens combining two NRTIs with DTG is preferred in our study cohort.

## Supporting information

S1 Table Frequency of TDRMs in the German HIV-1 Seroconverter Study Cohort (1996–2017) according to the Stanford HIVdb SDRM list. Mutations predicted by the Stanford HIVdb algorithm version 8.4 to result in potential low-level resistance are marked in gray.
(DOCX)
