# Supplementary material for: Prevalence and persistence of transmitted drug resistance mutations in the German HIV-1 Seroconverter Study Cohort
Source: PLoS One. 2019 Jan 16;14(1):e0209605. doi: 10.1371/journal.pone.0209605 (PMC6334938; doi:10.1371/journal.pone.0209605)
Supplement: S1 Table — Mutations predicted by the Stanford HIVdb algorithm version 8.4 to result in potential low-level resistance are marked in gray. (DOCX) [file pone.0209605.s001.docx]

**Table S1:** **Frequency of TDRMs in the** **German HIV-1 Seroconverter Study Cohort (1996-2017) according to the Stanford HIVdb SDRM-list**

| **Mutation** | **Prediction** | **Number of patients** | **Loss (N)** | **Longest observation (y)** | **Last observed exit (y)** | **Median (IQR) observation time (py)** |
| --- | --- | --- | --- | --- | --- | --- |
| **NNRTI** | | | | | | |
| A98G | Intermediate resistance | 8 | 0 | 6.5 | - | 0.2 (0.0-0.3) |
| L100I | High-level resistance | ~~-~~ | ~~-~~ | ~~-~~ | ~~-~~ | ~~-~~ |
| L100V | Intermediate resistance | ~~-~~ | ~~-~~ | ~~-~~ | ~~-~~ | ~~-~~ |
| K101E | Intermediate resistance | 1 | 0 | 0.0 | - | 0.0 (0.0-0.0) |
| K101H | Low-level resistance | ~~-~~ | ~~-~~ | ~~-~~ | ~~-~~ | ~~-~~ |
| K101P | High-level resistance | - | ~~-~~ | ~~-~~ | ~~-~~ | ~~-~~ |
| K103H | High-level resistance | - | - | - | - | - |
| K103N | High-level resistance | 54 | 3 | 6.6 | 6.6 | 0.0 (0.0-0.0) |
| K103S | High-level resistance | 3 | 0 | 2.0 | - | 0.7 (0.4-0.7) |
| K103T | High-level resistance | - | - | - | - | - |
| V106A | High-level resistance | 2 | 0 | 0.9 | - | 0.3 (0.1-0.4) |
| V106M | High-level resistance | - | - | - | - | - |
| V108I | Low-level resistance | 7 | 1 | 3.4 | 2.5 | 0.0 (0.0-0.0) |
| E138A | Low-level resistance | 73 | 1 | 9.3 | 9.3 | 0.0 (0.0-0.0) |
| E138G | Low-level resistance | 14 | 1 | 9.3 | 9.3 | 0.1 (0.1-0.2) |
| E138K | Intermediate resistance | 7 | 1 | 7.6 | 6.7 | 0.1 (0.0-0.2) |
| E138Q | Low-level resistance | 3 | 0 | 4.5 | - | 0.3 (0.0-1.5) |
| E138R | Low-level resistance | 1 | 0 | 0 | - | 0.0 (0.0-0.0) |
| V179D | Potential low-level resistance | 42 | 0 | 3.5 | - | 0.0 (0.0-0.0) |
| V179E | Potential low-level resistance | 13 | 0 | 1.7 | - | 0.0 (0.0-0.0) |
| V179F | Low-level resistance | 2 | 0 | 0.3 | - | 0.1 (0.0-0.1) |
| V179L | Low-level resistance | - | - | - | - | - |
| Y181C | High-level resistance | 6 | 0 | 3.4 | - | 0.1 (0.0-0.2) |
| Y181F | High-level resistance | - | - | - | - | - |
| Y181G | High-level resistance | - | - | - | - | - |
| Y181I | High-level resistance | - | - | - | - | - |
| Y181S | High-level resistance | 1 | 0 | 0.6 | - | 0.6 (0.6-0.6) |
| Y181V | High-level resistance | - | - | - | - | - |
| Y188C | High-level resistance | 1 | 1 | 3.3 | 1.5 | 3.0 (3.0-3.0) |
| Y188F | High-level resistance | - | - | - | - | - |
| Y188H | High-level resistance | - | - | - | - | - |
| Y188L | High-level resistance | 8 | 0 | 5.9 | - | 0.2 (0.1-0.3) |
| G190A | High-level resistance | 14 | 1 | 4.4 | 4.4 | 0.0 (0.0-0.1) |
| G190C | High-level resistance | - | - | - | - | - |
| G190E | High-level resistance | - | - | - | - | - |
| G190Q | High-level resistance | - | - | - | - | - |
| G190S | High-level resistance | 1 | 0 | 1.0 | - | 1.0 (1.0-1.0) |
| G190T | High-level resistance | - | - | - | - | - |
| G190V | High-level resistance | - | - | - | - | - |
| H221Y | Low-level resistance | 6 | 0 | 1.4 | - | 0.1 (0.1-0.1) |
| P225H | Intermediate resistance | 4 | 0 | 1.5 | - | 0.3 (0.2-0.4) |
| F227C | Intermediate resistance | - | - | - | - | - |
| F227L | Intermediate resistance | 1 | 0 | 0.3 | - | 0.3 (0.3-0.3) |
| M230I | Intermediate resistance | - | - | - | - | - |
| M230L | High-level resistance | - | - | - | - | - |
| K238N | Potential low-level resistance | 1 | 0 | 0.0 | - | 0.0 (0.0-0.0) |
| K238T | Intermediate resistance | - | - | - | - | - |
| Y318F | Intermediate resistance | - | - | - | - | - |
| N348I | Low-level resistance | - | - | - | - | - |
| **NRTI** | | | | | | |
| M41L | Low-level resistance | 55 | 1 | 6.6 | 6.6 | 0.0 (0.0-0.0) |
| K65E | Potential low-level resistance | - | - | - | - | - |
| K65N | Intermediate resistance | - | - | - | - | - |
| K65R | High-level resistance | 2 | 0 | 0.2 | - | 0.1 (0.0-0.1) |
| D67E | Potential low-level resistance | - | - | - | - | - |
| D67G | Potential low-level resistance | 4 | 0 | 4.6 | - | 0.4 (0.1-0.9) |
| D67H | Potential low-level resistance | - | - | - | - | - |
| D67N | Low-level resistance | 13 | 0 | 5.9 | - | 0.1 (0.0-0.1) |
| D67S | Potential low-level resistance | 1 | 0 | 0.5 | - | 0.5 (0.5-0.5) |
| D67T | Potential low-level resistance | - | - | - | - | - |
| D67del | Intermediate resistance | - | - | - | - | - |
| S68del | Intermediate resistance | - | - | - | - | - |
| T69D | Intermediate resistance | 2 | 0 | 1.0 | - | 0.4 (0.3-0.5) |
| T69G | Potential low-level resistance | - | - | - | - | - |
| T69ins | High-level resistance | - | - | - | - | - |
| T69del | Intermediate resistance | - | - | - | - | - |
| K70E | Low-level resistance | - | - | - | - | - |
| K70G | Low-level resistance | - | - | - | - | - |
| K70N | Low-level resistance | - | - | - | - | - |
| K70Q | Low-level resistance | - | - | - | - | - |
| K70R | Intermediate resistance | 9 | 0 | 6.9 | - | 0.1 (0.1-0.1) |
| K70S | Low-level resistance | - | - | - | - | - |
| K70T | Low-level resistance | 1 | 0 | 1.0 | - | 1.0 (1.0-1.0) |
| K70del | Intermediate resistance | - | - | - | - | - |
| L74I | High-level resistance | 2 | 1 | 1.5 | 1.5 | 0.8 (0.8-0.8) |
| L74V | High-level resistance | - | - | - | - | - |
| V75A | Intermediate resistance | - | - | - | - | - |
| V75M | Intermediate resistance | - | - | - | - | - |
| V75S | Intermediate resistance | - | - | - | - | - |
| V75T | High-level resistance | - | - | - | - | - |
| F77L | Potential low-level resistance | 15 | 0 | 10.2 | - | 0.2 (0.0-0.3) |
| Y115F | High-level resistance | 1 | 0 | 1.0 | - | 1.0 (1.0-1.0) |
| F116Y | Potential low-level resistance | - | - | - | - | - |
| Q151L | Intermediate resistance | - | - | - | - | - |
| Q151M | High-level resistance | - | - | - | - | - |
| M184I | High-level resistance | - | - | - | - | - |
| M184V | High-level resistance | 12 | 3 | 7.9 | 5.7 | 0.1 (0.0-0.1) |
| L210W | Low-level resistance | 15 | 2 | 3.4 | 3.4 | 0.1 (0.0-0.1) |
| T215A | Low-level resistance | 9 | 2 | 2.2 | 2.2 | 0.1 (0.0-0.1) |
| T215C | Low-level resistance | 9 | 2 | 4.8 | 4.8 | 0.1 (0.1-0.2) |
| T215D | Low-level resistance | 29 | 1 | 4.9 | 4.9 | 0.2 (0.1-0.2) |
| T215E | Low-level resistance | 9 | 0 | 5.9 | - | 0.1 (0.0-0.2) |
| T215F | Intermediate resistance | 2 | 0 | 0.0 | - | 0.0 (0.0-0.0) |
| T215I | Low-level resistance | 1 | 0 | 1.3 | - | 1.3 (1.3-1.3) |
| T215L | Low-level resistance | 3 | 0 | 0.9 | - | 0.1 (0.1-0.3) |
| T215N | Low-level resistance | 3 | 1 | 1.0 | 1.0 | 0.0 (0.0-0.0) |
| T215S | Low-level resistance | 50 | 2 | 5.5 | 5.0 | 0.3 (0.1-0.5) |
| T215V | Low-level resistance | - | - | - | - | - |
| T215Y | Intermediate resistance | 8 | 3 | 6.6 | 2.6 | 0.0 (0.0-0.1) |
| K219E | Potential low-level resistance | - | - | - | - | - |
| K219N | Potential low-level resistance | 2 | 0 | 0.3 | - | 0.1 (0.1-0.1) |
| K219Q | Potential low-level resistance | 19 | 0 | 5.9 | - | 0.1 (0.0-0.1) |
| K219R | Potential low-level resistance | 2 | 1 | 4.6 | 4.6 | 1.2 (0.0-2.3) |
| K219W | Potential low-level resistance | - | - | - | - | - |
| **PI** | | | | | | |
| L10F | Low-level resistance | 11 | 0 | 0.6 | - | 0.0 (0.0-0.1) |
| K20T | Low-level resistance | 3 | 1 | 1.3 | 1.3 | 0.1 (0.0-0.2) |
| L23I | Low-level resistance | 1 | 1 | 3.6 | 2.8 | 0.9 (0.9-0.9) |
| L24F | Potential low-level resistance | - | - | - | - | - |
| L24I | Low-level resistance | 3 | 0 | 1.0 | - | 0.3 (0.3-0.3) |
| L24M | Potential low-level resistance | - | - | - | - | - |
| D30N | High-level resistance | 3 | 0 | 1.0 | - | 0.1 (0.0-0.3) |
| V32I | Intermediate resistance | 2 | 0 | 0.5 | - | 0.2 (0.1-0.2) |
| L33F | Potential low-level resistance | 9 | 0 | 1.4 | - | 0.1 (0.0-0.1) |
| K43T | Potential low-level resistance | 4 | 1 | 2.2 | 2.2 | 0.2 (0.2-0.5) |
| M46I | Intermediate resistance | 13 | 2 | 8.5 | 4.3 | 0.5 (0.3-0.7) |
| M46L | Low-level resistance | 15 | 1 | 5.4 | 5.4 | 0.1 (0.0-0.2) |
| M46V | Low-level resistance | 3 | 2 | 6.4 | 6.4 | 0.1 (0.0-0.4) |
| I47A | High-level resistance | - | - | - | - | - |
| I47V | Intermediate resistance | 3 | 0 | 0.3 | - | 0.1 (0.0-0.1) |
| G48A | High-level resistance | - | - | - | - | - |
| G48L | High-level resistance | - | - | - | - | - |
| G48M | High-level resistance | - | - | - | - | - |
| G48Q | High-level resistance | - | - | - | - | - |
| G48S | High-level resistance | - | - | - | - | - |
| G48T | High-level resistance | - | - | - | - | - |
| G48V | High-level resistance | - | - | - | - | - |
| I50L | High-level resistance | - | - | - | - | - |
| I50V | High-level resistance | - | - | - | - | - |
| F53L | Low-level resistance | 2 | 0 | 0.0 | - | 0.0 (0.0-0.1) |
| I54A | Low-level resistance | - | - | - | - | - |
| I54L | High-level resistance | 3 | 0 | 5.9 | - | 0.3 (0.1-2.0) |
| I54M | High-level resistance | 1 | 0 | 0.0 | - | 0.0 (0.0-0.0) |
| I54S | Low-level resistance | - | - | - | - | - |
| I54T | Low-level resistance | - | - | - | - | - |
| I54V | Low-level resistance | 8 | 1 | 3.9 | 3.9 | 0.1 (0.1-0.2) |
| Q58E | Low-level resistance | 11 | 0 | 2.7 | - | 0.1 (0.0-0.2) |
| G73A | Low-level resistance | - | - | - | - | - |
| G73C | Low-level resistance | - | - | - | - | - |
| G73D | Potential low-level resistance | - | - | - | - | - |
| G73S | Low-level resistance | 2 | 0 | 0.5 | - | 0.1 (0.0-0.2) |
| G73T | Low-level resistance | 1 | 0 | 0.3 | - | 0.3 (0.3-0.3) |
| G73V | Potential low-level resistance | - | - | - | - | - |
| T74P | Low-level resistance | 1 | 0 | 0.0 | - | 0.0 (0.0-0.0) |
| L76V | High-level resistance | 1 | 0 | 0.1 | - | 0.1 (0.1-0.1) |
| V82A | Intermediate resistance | 10 | 0 | 3.9 | - | 0.0 (0.0-0.1) |
| V82C | Intermediate resistance | - | - | - | - | - |
| V82F | Intermediate resistance | 1 | 0 | 0.8 | - | 0.8 (0.8-0.8) |
| V82L | Intermediate resistance | 6 | 0 | 2.7 | - | 0.1 (0.1-0.2) |
| V82M | Intermediate resistance | - | - | - | - | - |
| V82S | Intermediate resistance | - | - | - | - | - |
| V82T | Intermediate resistance | - | - | - | - | - |
| N83D | Low-level resistance | - | - | - | - | - |
| I84A | High-level resistance | - | - | - | - | - |
| I84C | High-level resistance | - | - | - | - | - |
| I84V | High-level resistance | 4 | 0 | 1.3 | - | 0.1 (0.1-0.2) |
| N88D | High-level resistance | 3 | 0 | 1.0 | - | 0.1 (0.0-0.3) |
| N88G | Intermediate resistance | - | - | - | - | - |
| N88S | High-level resistance | - | - | - | - | - |
| N88T | Intermediate resistance | - | - | - | - | - |
| L89V | Potential low-level resistance | 2 | 0 | 0.5 | - | 0.2 (0.1-0.2) |
| L90M | High-level resistance | 22 | 0 | 8.4 | - | 0.0 (0.0-0.1) |

N: Number; py: Person years;
